# Knowledge, Attitude and Practice of Pilgrims Regarding Heat-Related Illnesses during the 2017 Hajj Mass Gathering

**DOI:** 10.3390/ijerph16173215

**Published:** 2019-09-03

**Authors:** Saber Yezli, Abdulaziz Mushi, Yara Yassin, Fuad Maashi, Anas Khan

**Affiliations:** 1 The Global Centre for Mass Gatherings Medicine, Ministry of Health, Riyadh 12341, Saudi Arabia; 2 Department of Emergency Medicine, College of Medicine, King Saud University, Riyadh 11472, Saudi Arabia

**Keywords:** Hajj, mass gathering, heat illnesses, health promotion, pilgrims, public health

## Abstract

The Hajj mass gathering attended by over two million Muslim pilgrims from around the world is a risk for heat-related illnesses (HRIs). We investigated the knowledge, attitude and practice (KAP) of pilgrims attending the 2017 Hajj regarding HRIs and their prevention. Adult pilgrims (1801) from six countries in Africa, Asia and the Middle East were interviewed using a structured questionnaire. Pilgrims had a mean age of 47.2 years (SD = 12.6) and a male:female ratio of 2.2:1. Over 83% declared having at least a secondary education. Pilgrims generally had good knowledge and above average attitude and practice according to our scoring criteria. Most pilgrims were aware of HRIs and preventive measures. However, poor hydration and reluctance to use certain preventive measures or to change Hajj activities’ schedule based on environmental temperature were noted. Age, nationality and level of education were significantly associated with a good knowledge of HRIs. Only nationality was significantly associated with good attitude, and good practice was significantly associated with gender, age and nationality. There were significant positive correlations between the KAP scores. These results can serve as baseline data to design effective general or targeted interventions to improve pilgrims’ knowledge and behavior and to reduce their risk of HRIs during Hajj.

## 1. Introduction

Exposure to a hot environment may result in a variety of heat-related illnesses (HRIs) of which heat exhaustion and heat stroke are the most serious and require immediate management [1,2]. The adverse effects of elevated temperature on human health have been widely described, including significant associated mortality [3]. For instance, in Europe, at least 70,000 excess fatalities were reported as a result of the 2003 summer heat wave, and 50,000 died in the Russian heat wave of 2010 [4,5]. With climate change, temperature-associated health impact is expected to be more pronounced in the future and will likely affect most populations [6].

The Hajj religious mass gathering presents many of the risk factors for HRIs. The event takes places in the hot and arid climate of Makkah, Kingdom of Saudi Arabia (KSA), and it attracts over two million Muslim pilgrims with different ethnicities and adaptations to heat, originating from up to 180 countries with varied climates [7]. Depending on the Islamic (lunar) calendar, Hajj may occur during the summer season, when temperatures in Makkah can reach 50 °C [8]. In addition to the climate, heat dissipated from Hajj activities—including crowds, human activities and mass vehicle exhaust—augments the heat load at the event [8]. The Hajj population and pilgrims’ behaviors can also predispose them to HRIs. A large proportion of pilgrims are elderly, and many have underlying health conditions, both of which are risk factors for HRIs [1]. Additionally, many pilgrims lack awareness of the seriousness of heat exposure and are overzealous in performing physically demanding religious rituals during the peak sunshine hours without umbrellas or head covering, with little food and with suboptimal hydration [8,9,10].

Heat-related illness are preventable and treatable, and various strategies are used to reduce the impact of these illnesses [2,11,12,13]. The Saudi authorities have significantly invested in the prevention of HRIs and in the optimization of their management during Hajj. These include improvements in the Hajj infrastructure and services, medical or otherwise, available for pilgrims. Umbrellas and water are freely available at the event, and HRIs-related health education campaigns are implemented pre and during each Hajj. Pilgrims’ attitude to risk, adaptation practices, avoiding risky behavior, and their knowledge regarding HRIs and early recognition of their symptoms are some of the most important factors in reducing the health impact of heat during Hajj. Hence, evaluating these factors is crucial to inform appropriate interventions for improvement. In the current study, we assessed the knowledge, attitude and practice (KAP) of pilgrims attending the 2017 Hajj regarding HRIs and their prevention.

## 2. Methods

### 2.1. Study Design, Setting and Population

This cross-sectional study was conducted in Makkah, Saudi Arabia, among adult (>18 years old) Hajj pilgrims from 6 countries (Table 1). The study was performed during the 2017 Hajj month from 23 August to 20 September. The sample size was calculated using a margin of error of 2.5%, a confidence interval (CI) of 95%, an approximate pilgrim population from the target countries of 600,000, and an expected response proportion of 50% to most of the main questions. The minimum sample size estimated for the study was 1533. By the end of the study, we enrolled a larger sample size of 1801 pilgrims to account for errors and non-respondents.

### 2.2. Survey Design and Scoring System

Data were collected using an anonymous structured questionnaire administered through trained investigators. The questionnaire was designed to collect KAP information concerning heat-related illness and their prevention as well as collecting participants’ demographics, including age, gender, nationality and level of education. The questionnaire was developed in English by reviewing available surveys in the literature [14,15,16,17,18], but it was tailored for the Hajj setting and the study objectives. The questionnaire’s content was then reviewed by two public health experts with Hajj experience and face-validated by piloting it among 24 pilgrims before the study with feedback for doubtful or confusing items. The Cronbach’s alpha for the knowledge, attitude and practice sections of the questionnaire were 0.72, 0.78 and 0.82, respectively, and were deemed acceptable.

A scoring system was developed to score the KAP responses as described previously [19]. Briefly, incorrect/inappropriate or uncertain (don’t know) responses were given a 0 score, while a score of 1 was given for choosing the correct/appropriate answer; a correct/appropriate response being that based on current literature and best practice. For multiple choice questions with more than one correct answer, 1 score was given for choosing the correct/appropriate response and for not choosing the incorrect/inappropriate responses. The score for the question was then divided by the total number of multiple choices in the question to standardize the scores to be between 0 and 1. Additionally, overall mean scores, ranging from 0 to 1, for each section of the questionnaire (knowledge, attitude and practice) were calculated and were then further divided into 4 categories to reflect the level of KAP among pilgrims. These were: Poor (score <0.25), below average (0.25 ≤ score < 0.50), above average (0.5 ≤ score < 0.75), and good (score ≥0.75).

### 2.3. Statistical Analysis

Descriptive statistics such as the mean and standard deviation (SD) were computed for quantitative variables, and frequencies and percentages were calculated for categorical variables. Cronbach’s α was used to measure the reliability and internal consistency for the KAP questions. Differences in means were evaluated using the t-test. The difference of KAP scores with respect to individual covariates was evaluated by the Mann–Whitney U test or Kruskal–Wallis test, as appropriate. Binary logistic regression was used to compute odds ratios with 95% confidence intervals and to assess the presence and degree of association between the dependent versus independent variables. Variables having a *p*-value <0.05 at the bi-variable analysis were taken for multivariate analysis. The correlation between knowledge attitude and practice was examined using the Spearman correlation coefficient. All of the tests for significance were two-sided, and *p* values <0.05 were considered statistically significant. All analyses were done using the SPSS 22.0 (SPSS Inc., Chicago, IL, USA) software program.

### 2.4. Ethics and Confidentiality

All study participants were briefed about the study and gave verbal consent before enrolment. The study was approved by the King Fahad Medical City Ethics Committee and the Institutional Review Board (IRB log: 17-304E), and it was conducted following the Ethics Committee’s guidelines. The KAP survey forms were anonymous and did not include any identifiers or personal information of the participants.

## 3. Results

### 3.1. Characteristics of the Study Population

The study enrolled 1801 pilgrims, nationals of six countries (two from Asia, two from Africa, and two from Arab countries in the Middle East). The characteristics of the study population are summarized in Table 1. The mean age of the participants was 47.2 years (SD = 12.6, range = 19–96 years) with a male:female ratio of 2.2:1. Pilgrims from Egypt were the least represented (5.9%), and most pilgrims (>83%) declared having at least a secondary education.

### 3.2. Knowledge

Most pilgrims (81.5%) declared that they were aware of the Hajj season’s weather conditions in Saudi Arabia before traveling to the Kingdom, and 65.7% had received health information about HRIs either before travel (52.7%), upon arrival to KSA (11.9%) or both (1.1%). Over 85% knew that there is free water available for pilgrims at the Hajj holly sites, and 62% believed that they should be drinking 2–4 L of water a day during the Hajj (Supplementary Table S1). There was a statistically significant difference in the mean response between males and females (3.23 vs. 3.35 L, *p* = 0.007). The mean scores for each knowledge question are presented in Table 2. According to our scale, pilgrims had good knowledge regarding heat-related illness (overall mean knowledge score = 0.77, SD = 0.18), and 80% of them had a mean score of ≥0.75 (good knowledge). However, important knowledge gaps were noted. For example, 46.4% of pilgrims thought that being thirsty is the only sign of needing to drink water, and only 66% knew that sunscreen is protective. Additionally, although over 70% of pilgrims recognized dizziness and feeling tired and weak as signs of HRIs, smaller proportions recognized other symptoms such as feeling confused (45.6%) and muscle cramps (27.3%). In general, only 4% of pilgrims correctly identified all the HRIs symptoms in the questionnaire. Additionally, 18%–23% of pilgrims did not know that a high atmospheric temperature could cause illness or death and that older pilgrims or those with underlying health conditions were more likely to develop HRIs.

### 3.3. Attitude

The attitude of pilgrims towards HRIs was deemed above average (overall mean attitude score = 0.57, SD = 0.23) with only 34% having a mean score of ≥0.75 (good attitude). The lowest attitude scores were noted in relation to pilgrims changing their Hajj rituals schedule due to environmental temperature or level of crowdedness (Table 2). Over 85% of pilgrims would perform their Hajj rituals following their Hajj mission schedule even if the environment was overcrowded, and if it became extremely hot, only 35.5% would postpone Hajj rites until the conditions were cooler. Around 67% of pilgrims were willing to buy an umbrella and use it in unshaded areas, but only 48% were willing to use sunscreen while performing Hajj during the day. A quarter of pilgrims declared that they would not drink more water during a hot day if they were not thirsty, and 28% reported preferring to drink soft drinks and coffee when they feel thirsty.

### 3.4. Practice

As shown in Table 2, pilgrims reported an above average practice (overall mean practice score = 0.58, SD = 0.22), with only 31% having a mean score of ≥0.75 (good practice). Though 89% declared they dress based on weather conditions, only 43% check the weather forecast before going out. Similarly, <40% of pilgrims reported using sunscreen during Hajj or generally while doing outside activities during sunny days. Nearly half of pilgrims reported drinking water only when they feel thirsty, but 32.3% reported drinking soft drinks or coffee when they felt thirsty during Hajj. Most pilgrims (66.2%) reported drinking 2–4 L of water a day during Hajj, while nearly 10% drank <2 L. There was no statistically significant difference in the mean daily water intake among male and female pilgrims (2.99 vs. 3.00 L, *p* = 0.09). Around 60% of pilgrims reported using umbrellas in unshaded areas while performing Hajj, and 77.6% do walk in shaded roads even if it means their journey will take longer.

### 3.5. Demographic Variables and KAP Scores

There was a statistically significant difference in KAP scores in relation to gender, age groups, nationality and level of education (Table 3). Females had lower knowledge scores than males, but they had higher attitude and practice scores. Pilgrims aged 48–57 years had the highest knowledge and practice scores, but their attitude score was lower than those aged ≤47 years. Pilgrims from Indonesia had the highest attitude and practice scores and the second highest knowledge score. Pilgrims from Iraq, on the other hand, while having the highest knowledge score, had the lowest practice score. KAP scores increased with increasing level of education.

A multivariate analysis identified several independent predictors for having a good KAP (Table 4). Age, nationality and level of education were significantly associated with good knowledge of HRIs. Pilgrims aged 48–57 were two-times more likely to have good knowledge compared to those aged <38 years (adjusted odd ratio [adOR] = 2.02; 95% CI = 1.45–2.83). With the exception of pilgrims from Pakistan, pilgrims from other countries were 1.5–5.5 times more likely to have good knowledge compared to those from South Africa (Table 4). Similarly, pilgrims with secondary and higher educations were, respectively, 5.2 and 7.4 times more likely to have better knowledge than those with no formal education. Only nationality was significantly associated with good attitude. Pilgrims from Indonesia were 2.6 times more likely to have good attitude compared to those from South Africa (adOR = 2.6; 95% CI = 1.79–3.76). Pilgrims from other nationalities were less likely to have good attitude compared to South African pilgrims. Good practice was significantly associated with gender, age and nationality. Females were more likely to have good practice, and, compared to pilgrims aged >38 years old, other age groups were also more likely to have good practice. Unlike pilgrims from other nationalities, Indonesian pilgrims were more likely to have good practice compared to those from South Africa (adOR = 3.2; 95% CI = 2.18–4.73).

There was a weak but statistically significant positive correlation between knowledge and attitude (r_s_= 0.17, *p* < 0.0001) and knowledge and practice (r_s_ = 0.18, *p* < 0.0001). A significant positive moderate correlation was found between attitude and practice (r_s_ = 0.58, *p* < 0.0001).

## 4. Discussion

The Hajj religious mass gathering, currently taking place in the summer season, is a risk for HRIs. The latter are regularly reported during the event, causing significant morbidity and mortality [8,20]. KSA authorities have introduced numerous public health interventions to reduce the impact of environmental heat on Hajj pilgrims, including a substantial investment in health education and awareness campaigns regarding the issue. An important step in informing appropriate policies and health messages for pilgrims is understanding pilgrims’ KAP regarding HRIs and preventive measures. We report on the results of the first study to investigate the latter among pilgrims from six ethnically, culturally, and climatically varied countries. We found that pilgrims generally had good knowledge and above average attitude and practice according to our scoring criteria. However, some important gaps in knowledge, as well as negative attitudes and wrong practices, were also noted. Similar observations were reported in studies investigating KAP regarding heat-illness and heat waves among Arab Hajj pilgrims and other communities internationally [14,15,16,17,18,21].

We found that most pilgrims (>80%) appear to know about Hajj weather conditions and the availability of free water during the event before traveling to KSA. A study among Arab pilgrims also reported that 80.6% were aware of Makkah’s climate before arrival, and that there was a strong link between the extent to which pilgrims were aware of Makkah’s weather and dressing according to hot weather conditions [21]. In the current study, most pilgrims knew that dark colored clothes were not appropriate in hot weather and declared that they dress based on weather conditions. A similar high-level of awareness regarding the importance of clothing type in relation to heat-illness was reported among Californian hired farm workers [15] but not among the general population in Lisbon and Madrid [17].

The knowledge of symptoms associated with HRIs is important for timely actions to reduce the impact of such conditions or avoiding them worsening. A study among Arab pilgrims found that most reported experiencing some manifestations of high temperature during Hajj [21]. Over 50% of pilgrims considered elevated body temperature, excessive sweating, dizziness, fatigue, and headaches to be the main symptoms of problematic heat exposure. In the current study, over 70% of pilgrims recognized dizziness, feeling tired, and feeling weak as signs of heat-illnesses, but smaller proportions recognized other symptoms such as feeling confused (45.6%) and muscle cramps (27.3%). Studies from other settings have reported that the recognition of heat-illness symptoms varied depending on the symptoms and the population investigated. Fatigue, dehydration, headaches and dizziness were the most commonly recognized symptoms [14,16,17].

We report that most pilgrims were aware of some of the protective measures against HRIs; however, they were willing to use some measures more than others. For example, over 60% of pilgrims were willing to buy and use an umbrella during Hajj or reported using one during the event. This is in contrast to the much lower proportion of pilgrims willing to use or using sunscreen in Hajj, despite knowing that it is protective. Several factors could explain these observations, including cultural and religious beliefs. For instance, some pilgrims believe that certain actions such as wearing hats, using umbrellas or adding chemicals on the body are not compatible with the Hajj rituals. Education on these issues and including the involvement of religious and community leaders is important to address such misconceptions. The origin of the pilgrims and their common practices could also be a factor. For instance, pilgrims originating from countries where exposure to the sun is more common, such as Egypt and Iraq, were the least likely to use sunscreen. Differences in pilgrims’ behavior related to preventing HRIs according to country of origin have been reported previously [9,21].

Proper hydration is a basic method to prevent HRIs, and the risk of all forms of heat illness is greatly exacerbated by poor hydration [22]. The Adequate Intake (AI) volumes of water for adult men and adult non-pregnant/lactating women were reported to be 2.5–3.7 and 2–2.7 L/day, respectively [23]. These represent the amounts of water intake, from all sources, that should meet the needs of almost everyone in that specific life-stage/gender group who is healthy, consumes an average diet, and performs moderate levels of physical activity [23]. Given the characteristics of the Hajj and its population, it is expected that the AI volumes for pilgrims would be significantly higher than those listed above. We note that pilgrims largely both underestimated the amount of water they should be drinking and reported drinking less than adequate amounts during Hajj. This was despite most being aware of the importance of hydration, the effect of sweating, the need to drink more water when perfuming Hajj in hot weather, and the availability of free water at the holy sites during Hajj. Pilgrims reported drinking a mean of 3 L of water per day, which is slightly higher than the 2.7 L/day reported among pilgrims in 1997 [9]. Male pilgrims believed they needed significantly less water than females, which is concerning given that males, in general, require more water intake than females [23]. Other studies among populations at risk of HRIs reported similar findings, noting low or inadequate water consumption [15,18,24,25]. It is crucial that pilgrims are educated on the importance of proper hydration and are encouraged to drink enough water during Hajj, as dehydration not only increases their risk of HRIs but also increases the risk of other health complications, especially for those elderly with underlying health conditions such as diabetes and kidney diseases.

An important observation from the attitude questions was that most pilgrims were unwilling to change their Hajj plans based on environmental temperature or crowdedness and would follow their Hajj missions’ schedules regardless of these factors. In general, pilgrims come to Hajj with dedicated Hajj missions that organize their pilgrimage. It is therefore understandable that pilgrims would not be willing to go against their Hajj missions’ planned schedules. However, it is important that, whenever possible, Hajj missions plan activities so that to minimize the risk of heat illness and discourage pilgrims, especially vulnerable groups, from risky or unnecessary activities during hot times of the day. Venturing outside at noon during Hajj was reported to be common among pilgrims, with over half of those questioned stating that they do so sometimes or always [21].

There was a statistically significant difference in KAP scores in relation to gender, age groups, nationality and level of education. The latter was an independent predictor for having good knowledge but not good attitude or practice. Level of education has been liked to better knowledge and practice regarding HRIs and preventive measures both among Hajj pilgrims and other populations. Al-Mayahi and Ali Kabbash [21] found that during Hajj, pilgrims with a high-level of education had a higher water intake and appeared more inclined to stay within their residence at noon, when the heat is extremely high. They reasoned that these observations may indicate that a higher level of education is associated with a greater awareness of risks and better compliance with appropriate coping strategies. Studies related to heat waves and the impact of climate change among participants from China, Ethiopia, Belgium and the Netherlands all found that the level of knowledge of respondents increased with increasing levels of education [14,26,27].

We found that male pilgrims had significantly better knowledge regarding HRIs and their prevention than female pilgrims, but they had worst attitude and practice. Studies from the USA and China reported similar observations [14,15]. For instance, among the public in China, males were more knowledgeable regarding heat waves, but females had better practice. The authors reasoned that differences in risk awareness between the genders and the greater impact of heat on woman compared to man may explain this difference [14]. We also found that older age was significantly associated with good knowledge and practice relating to HRIs and their prevention. This is significant given that elderly people are more likely to develop HRIs and die as a consequence [28,29,30]. A study among Arab pilgrims reported that pilgrims over 40 years old showed better health-related practices, including a greater use of head cover and drinking more water [21]. However, this group of pilgrims also put themselves at an increased risk of heat-related outcomes by going out at noon and visiting the holy mosque more often during Hajj.

There was a statistically significant positive correlation between knowledge and attitude, knowledge and practice, and attitude and practice. A similar correlation was found in a KAP study related to heat waves among the public in China [14]. This correlation suggests that better knowledge about HRIs among pilgrims translated to a better attitude, which in turn was associated with better practice. However, the association between knowledge and attitude and knowledge and practice in our study was weak. Reports in the literature including from KAPs among Hajj pilgrims support the notions that there is no simple relationship between the level of knowledge, attitude and behaviors and that good knowledge does not always translate to better attitude or good practice. Hence, in addition to improving knowledge, it is important that interventions aimed at reducing the risk of HRIs during Hajj should be aimed at modifying pilgrims’ behavior in order to have an impact on practice.

Studies have shown that those individuals with low knowledge of HRIs and/or those who do not undertake adaptation and prevention practices are at increased risk of these illnesses [10,14]. Education and health awareness interventions are effective in improving knowledge, promoting positive attitude, and promoting good practice in relation to HRIs and their prevention [16,18,31]. Hajj pilgrims receive health education messages while they are in Saudi Arabia for Hajj, but many also have such campaigns administered in their country of origin before the pilgrimage. Such campaigns should include education on HRIs and their prevention, and they should address common misconceptions as well as believes and practices that increase the risk of HRIs among pilgrims. The engagement of Hajj authorities, health providers, travel agents, pilgrims’ health missions and community and religious leaders in this endeavor is important. In this context, we report that around 65% of pilgrims declared receiving health information regarding the topic, and most was pre-arrival. Such interventions may partially be responsible for the high awareness of pilgrims regarding HRIs found in our study, as >80% of pilgrims recognized that high atmospheric temperatures cause specific illnesses. This in contrast to a 1995 study where only 26% of pilgrims knew about HRIs [10]. While this is encouraging, efforts should be dedicated to ensure that all pilgrims receive appropriate heat-related health information, including on the availability of free umbrellas and water during Hajj [32]. This is especially important given the Hajj has entered the hot season and will continue taking place in the hot months for many years to come. As pilgrims originate from countries with different cultures, languages, believes, educational, and health system backgrounds, as well as climate conditions, health education materials should be developed taking into account these factors. They should be administered through the most appropriate means for maximum efficacy both pre- and post-arrival to KSA. Targeted interventions tailored for specific risk groups may also be necessary.

Our study has some limitations. While we enrolled a large number of pilgrims from different countries, the sample size represents a small proportion of the overall Hajj population at large. The latter limitation, in addition to the cross-sectional design of the study, limits the generalizability of the findings. Additionally, data were collected using a questionnaire; therefore, responses obtained were prone to information bias. Similarly, we did not observe practice; hence, the results may not accurately reflect actual practice among pilgrims.

## 5. Conclusions

This is the first attempt to study KAP relating to HRIs and their prevention among Hajj pilgrims from countries with large pilgrim populations which vary ethnically, culturally, and climatically. We found that pilgrims generally had good knowledge and above average attitude and practice according to our scoring criteria. However, we identified knowledge gaps and poor attitudes and practices, which can serve as baseline data to design effective general or targeted interventions to address these shortcomings. Further studies at larger scales including qualitative methods and observation of actual practices are warranted.

## Figures and Tables

**Table 1:** Characteristics of the study population.

Variable	Category	*n*	%
Gender		1769	
	Male	1221	69.0
	Female	548	31.0
Age (years)		1790	
	<38	444	24.8
	38–47	465	26.0
	48–57	462	25.8
	>57	419	23.4
Nationality		1788	
	South Africa	304	17.0
	Nigeria	397	22.2
	Pakistan	299	16.7
	Egypt	105	5.9
	Iraq	477	26.7
	Indonesia	206	11.5
Level of education		1750	
	No formal education	97	5.5
	Primary education	188	10.7
	Secondary education	668	38.2
	University/higher education	797	45.5

**Table 2:** Mean hear-related illness knowledge, attitude and practice scores.

Statement/Question	*N*	Min	Max	Mean	SD
Knowledge					
Do you believe that high atmospheric temperatures cause specific illnesses?	1795	0	1	0.81	0.39
Which of the following could be symptoms for heat illnesses?	1794	0	1	0.65	0.16
Excessive sweating causes loss in body fluids components such as minerals.	1789	0	1	0.82	0.39
Wearing dark-colored clothes are better during hot weather	1792	0	1	0.74	0.44
Being thirsty is the only sign of needing to drink water	1788	0	1	0.51	0.50
Using sunscreen reduces the risk of HRIs.	1795	0	1	0.66	0.47
Performing Hajj rites in hot weather lead to the need to drink more water.	1793	0	1	0.97	0.18
Pilgrims with underlying health conditions (e.g., Diabetes, Hypertension) are more likely to develop HRIs.	1792	0	1	0.78	0.42
Older pilgrims more likely to develop HRIs.	1792	0	1	0.82	0.38
High atmospheric temperatures can cause death.	1794	0	1	0.77	0.42
Exposure to the sunlight during hot weather leads to fever	1797	0	1	0.71	0.45
Good ventilation plays a role in cooling the atmosphere	1796	0	1	0.91	0.28
Overcrowding plays a role in increasing atmospheric temperatures	1794	0	1	0.88	0.33
Overall	1801	0	1	0.77	0.18
Attitude					
Would you drink more water during a hot day even if you are not thirsty?	1799	0	1	0.75	0.43
Would you use an umbrella in unshaded areas while performing Hajj rites?	1775	0	1	0.67	0.47
While performing Hajj, if it became extremely hot, would you postpone your rites until it becomes cooler?	1631	0	1	0.40	0.49
If possible, will you perform Hajj rites at night?	1650	0	1	0.70	0.46
Even if it is overcrowded, I will perform the Hajj rites following my mission schedule.	1777	0	1	0.13	0.34
Are you willing to pay for an umbrella?	1748	0	1	0.70	0.46
Would you use a sunscreen while performing Hajj rites during the day?	1729	0	1	0.50	0.50
I prefer to drink soft drinks or coffee when I feel thirsty	1720	0	1	0.71	0.46
Overall	1801	0	1	0.57	0.23
Practice					
I dress based on the weather conditions	1787	0	1	0.89	0.31
I walk in a shaded road even if it takes longer	1786	0	1	0.78	0.42
I use an umbrella in unshaded areas while performing Hajj rites	1768	0	1	0.61	0.49
I drink water only when I feel thirsty	1792	0	1	0.52	0.50
I check for the weather forecast /bulletin before going out	1790	0	1	0.43	0.49
I drink soft drinks or coffee when I feel thirsty during Hajj	1746	0	1	0.67	0.47
I use sunscreen while performing Hajj rites	1745	0	1	0.36	0.48
Besides Hajj season, do you use a sunscreen in outdoor activities during a sunny day?	1754	0	1	0.40	0.49
Overall	1801	0	1	0.58	0.22

Min: Minimum; Max: Maximum; SD: Standard deviation; *N*: Number of observations.

**Table 3:** Demographic variables and knowledge, attitude and practice scores.

Variable	Category	*N*	Knowledge Scores			Attitude Scores			Practice Scores		
			Mean	SD	*p*-Value *	Mean	SD	*p*-Value *	Mean	SD	*p*-Value *
Gender					0.036			<0.0001			0.001
	Male	1221	0.77	0.17		0.55	0.24		0.57	0.21	
	Female	548	0.76	0.18		0.61	0.22		0.60	0.23	
Age (years)					<0.0001			<0.0001			<0.0001
	<38	444	0.74	0.18		0.61	0.22		0.56	0.24	
	38–47	465	0.78	0.18		0.60	0.21		0.61	0.21	
	48–57	462	0.79	0.17		0.58	0.20		0.62	0.19	
	>57	419	0.75	0.18		0.49	0.27		0.52	0.22	
Nationality					<0.0001			<0.0001			<0.0001
	South Africa	304	0.70	0.18		0.67	0.15		0.63	0.21	
	Nigeria	397	0.75	0.17		0.60	0.24		0.56	0.22	
	Pakistan	299	0.69	0.16		0.59	0.18		0.55	0.21	
	Egypt	105	0.78	0.20		0.40	0.24		0.57	0.26	
	Iraq	477	0.85	0.15		0.43	0.21		0.51	0.17	
	Indonesia	206	0.82	0.14		0.75	0.18		0.72	0.22	
Level of education					<0.0001			<0.0001			<0.0001
	No formal education	97	0.64	0.19		0.47	0.28		0.44	0.23	
	Primary education	188	0.69	0.18		0.49	0.29		0.52	0.24	
	Secondary education	668	0.76	0.17		0.58	0.21		0.59	0.20	
	University/Higher education	797	0.81	0.16		0.59	0.22		0.60	0.21	

* *p*-value for the Mann–Whitney U or Kruskal–Wallis test; *N*: Number of observations; SD: Standard deviation.

**Table 4:** Association between variables and good knowledge, attitude and practice.

Variable	*N*	Good Knowledge		Good Attitude		Good Practice	
		OR [95%]	aOR [95%]	OR [95%]	aOR [95%]	OR [95%]	aOR [95%]
Gender							
Female	548	1	1	1		1	1
Male	1221	1.25 (1.02–1.53) *	0.90 (0.70–1.14)	0.86 (0.69–1.06)		0.56 (0.45–0.70) *	0.63 (0.49–0.80) *
Age (years)							
<38	444	1	1	1	1	1	1
38–47	465	1.45 (1.11–1.88) *	1.19 (0.88–1.61)	0.92 (0.70–1.20)	1.19 (0.89–1.60)	1.28 (0.97–1.69)	1.56 (1.15–2.13) *
48–57	462	2.21 (1.68–2.91) *	2.02 (1.45–2.83) *	0.68 (0.51–0.89) *	0.99 (0.73–1.35)	1.15 (0.87–1.52)	1.68 (1.21–2.34) *
>57	419	1.35 (1.03–1.77) *	1.23 (0.84–1.79)	0.52 (0.39–0.70) *	0.99 (0.71–1.40)	0.59 (0.43–0.80) *	1.02 (0.69–1.52)
Nationality							
South Africa	304	1	1	1	1	1	1
Nigeria	397	1.26 (0.93–1.70)	1.50 (1.05–2.14) *	0.70 (0.52–0.95) *	0.70 (0.51–0.96) *	0.73 (0.53–1.00)	0.79 (0.55–1.13)
Pakistan	299	0.53 (0.39–0.74) *	0.54 (0.38–0.78) *	0.87 (0.63–1.21)	0.87 (0.63–1.21)	0.51 (0.36–0.72) *	0.64 (0.44–0.92) *
Egypt	105	2.03 (1.28–3.24) *	2.88 (1.70–4.88) *	0.21 (0.11–0.37) *	0.21 (0.11–0.38) *	0.74 (0.47–1.19)	0.83 (0.50–1.37)
Iraq	477	4.25 (3.08–5.86) *	5.51 (3.78–8.02) *	0.12 (0.08–0.18) *	0.12 (0.08–0.18) *	0.24 (0.17–0.34) *	0.26 (0.18–0.39) *
Indonesia	206	2.67 (1.82–3.92) *	3.17 (2.10–4.78) *	2.62 (1.81–3.79) *	2.60 (1.79–3.76) *	2.77 (1.92–4.00) *	3.21 (2.18–4.73) *
Level of education							
No formal education	97	1	1	1		1	1
Primary education	188	1.57 (0.94–2.62)	1.45 (0.83–2.53)	1.13 (0.66–1.94)		1.43 (0.80–2.58)	1.11 (0.59–2.10)
Secondary education	668	3.21 (2.04–5.03) *	5.21 (3.09–8.78) *	1.43 (0.90–2.30)		1.72 (1.02–2.88) *	1.18 (0.66–2.10)
University/Higher education	797	4.48 (2.87–7.04) *	7.39 (4.37–12.5) *	1.28 (0.80–2.05)		1.93 (1.15–3.22) *	1.55 (0.88–2.73)

OR: Odd ratio; aOR: Adjusted odd ratio; * Statistically significant.

## Supplementary Materials

The following are available online at https://www.mdpi.com/1660-4601/16/17/3215/s1, Table S1: Knowledge, attitude and practice of pilgrims regarding heat-related illnesses.
