# Supplementary material for: Knowledge, Attitude and Practice of Pilgrims Regarding Heat-Related Illnesses during the 2017 Hajj Mass Gathering
Source: Int J Environ Res Public Health. 2019 Sep 3;16(17):3215. doi: 10.3390/ijerph16173215 (PMC6747626; doi:10.3390/ijerph16173215)
Supplement: Supplementary file 1 [file ijerph-16-03215-s001.pdf]

Table S1. Knowledge, attitude and practice of pilgrims regarding heat-related illnesses

Variable	Category	*n*	%
**Knowledge questions**			
Do you believe that high atmospheric temperatures cause specific illnesses?		1795	
	No	115	6.4
	Yes	1451	80.8
	Don't Know	229	12.8
Did you know what the weather conditions are during the current Hajj season, before traveling to Saudi Arabia?		1773	
	No	328	18.5
	Yes	1445	81.5
Have you received any health-related information about "Heat-related Illnesses"?		1786	
	No	612	34.3
	Yes before travel	942	52.7
	Yes upon arrival	212	11.9
	Yes before and upon arrival	20	1.1
Which of the following could be symptoms for heat illnesses?		1794	
	Bloody urine	163	9.1
	Feeling confused	818	45.6
	Muscle cramps	490	27.3
	Abdominal pain	386	21.5
	Visual disturbances/Blurred vision	832	46.4
	Feeling tired or weak	1279	71.3
	Cough with blood	127	7.1
	Dizziness	1258	70.1
Excessive sweating causes loss in body fluids components such as minerals.		1789	
	No	122	6.8
	Yes	1463	81.8
	Don't Know	204	11.4
Wearing dark-colored clothes are better during hot weather		1792	
	No	1322	73.8
	Yes	321	17.9
	Don't Know	149	8.3
Being thirsty is the only sign of needing to drink water		1788	
	No	912	51.0
	Yes	829	46.4
	Don't Know	47	2.6

How much water should you drink daily during the Hajj?		1741	
	<1.5	54	3.1
	1.5-<2	42	2.4
	2-<3	478	27.5
	3-<4	601	34.5
	4-<5	262	15.0
	5-<6	199	11.4
	6<10	94	5.4
	≥10	11	0.6
Do you know there is free water in holy sites?		1792	
	No	267	14.9
	Yes	1525	85.1
Using sunscreen reduces the risk of heat-related illnesses.		1795	
	No	349	19.4
	Yes	1183	65.9
	Don't Know	263	14.7
Performing Hajj rites in hot weather lead to the need to drink more water.		1793	
	No	18	1.0
	Yes	1734	96.7
	Don't Know	41	2.3
Pilgrims with underlying health conditions (e.g. Diabetes, Hypertension) are more likely to develop heat-related illnesses.		1792	
	No	121	6.8
	Yes	1394	77.8
	Don't Know	277	15.5
Older pilgrims are more likely to develop heat-related illnesses.		1792	
	No	103	5.7
	Yes	1471	82.1
	Don't Know	218	12.2
High atmospheric temperatures can cause death.		1794	
	No	109	6.1
	Yes	1385	77.2
	Don't Know	300	16.7
Exposure to the sunlight during hot weather leads to fever		1797	
	No	234	13.0
	Yes	1279	71.2
	Don't Know	284	15.8
Good ventilation plays a role in cooling the atmosphere		1796	

	No	84	4.7
	Yes	1639	91.3
	Don't Know	73	4.1
Overcrowding plays a role in increasing atmospheric temperatures		1794	
	No	119	6.6
	Yes	1577	87.9
	Don't Know	98	5.5
**Attitude questions**			
Would you drink more water during a hot day even if you are not thirsty?		1799	
	No	444	24.7
	Yes	1355	75.3
Would you use an umbrella in unshaded areas while performing Hajj rites?		1794	
	No	578	33.2
	Yes	1197	66.7
	Not Applicable	19	1.1
While performing Hajj, if it became extremely hot, would you postpone your rites until it becomes cooler?		1794	
	No	976	54.4
	Yes	655	36.5
	Not Applicable	163	9.1
If possible, will you perform Hajj rites at night?		1795	
	No	488	27.2
	Yes	1162	64.7
	Not Applicable	145	8.1
Even if it is overcrowded, I will perform the Hajj rites following my mission schedule		1793	
	No	238	13.3
	Yes	1539	85.5
	Not Applicable	16	0.9
Are you willing to pay for an umbrella?		1792	
	No	526	29.4
	Yes	1222	68.2
	Not Applicable	44	2.5
Would you use a sunscreen while performing Hajj rites during the day?		1790	
	No	871	48.7
	Yes	858	47.9
	Not Applicable	61	3.4
I prefer to drink soft drinks or coffee when I feel thirsty		1788	

	No	1216	68.0
	Yes	504	28.2
	Not Applicable	68	3.8
**Practice questions**			
I dress based on the weather conditions		1787	
	No	198	11.1
	Yes	1589	88.9
I walk in a shaded road even if it takes longer		1786	
	No	400	22.4
	Yes	1386	77.6
I use an umbrella in unshaded areas while performing Hajj rites		1789	
	No	696	38.9
	Yes	1072	59.9
	Not Applicable	21	1.2
I drink water only when I feel thirsty		1792	
	No	935	52.2
	Yes	857	47.8
My daily intake of water during Hajj (in Liters)		1730	
	<1.5	86	5.0
	1.5-<2	75	4.3
	2-<3	532	30.8
	3-<4	613	35.4
	4-<5	245	14.2
	5-<6	122	7.1
	6<10	44	2.5
	≥10	13	0.8
I check for the weather forecast /bulletin before going out		1790	
	No	1025	57.3
	Yes	765	42.7
I drink soft drinks or coffee when I feel thirsty during Hajj		1793	
	No	1166	65.0
	Yes	580	32.3
	Not Applicable	47	2.6
I use sunscreen while performing Hajj rites		1795	
	No	1122	62.5
	Yes	623	34.7
	Not Applicable	50	2.8

Besides Hajj season, do you use a sunscreen in outdoor activities during a sunny day?		1795	
	No	1055	58.8
	Yes	699	38.9
	Not Applicable	41	2.3
